# High-Throughput Large-Scale Targeted Proteomics Assays for Quantifying Pathway Proteins in *Pseudomonas putida* KT2440

**DOI:** 10.3389/fbioe.2020.603488

**Published:** 2020-12-02

**Authors:** Yuqian Gao, Thomas L. Fillmore, Nathalie Munoz, Gayle J. Bentley, Christopher W. Johnson, Joonhoon Kim, Jamie A. Meadows, Jeremy D. Zucker, Meagan C. Burnet, Anna K. Lipton, Aivett Bilbao, Daniel J. Orton, Young-Mo Kim, Ronald J. Moore, Errol W. Robinson, Scott E. Baker, Bobbie-Jo M. Webb-Robertson, Adam M. Guss, John M. Gladden, Gregg T. Beckham, Jon K. Magnuson, Kristin E. Burnum-Johnson

**Affiliations:** ^1^Department of Energy, Agile BioFoundry, Emeryville, CA, United States; ^2^Pacific Northwest National Laboratory, Richland, WA, United States; ^3^National Renewable Energy Laboratory, Golden, CO, United States; ^4^Sandia National Laboratories, Livermore, CA, United States; ^5^Oak Ridge National Laboratory, Oak Ridge, TN, United States

**Keywords:** targeted proteomics, *Pseudomonas putida* KT2440, mass spectrometry, selected reaction monitoring, central carbon metabolism

## Abstract

Targeted proteomics is a mass spectrometry-based protein quantification technique with high sensitivity, accuracy, and reproducibility. As a key component in the multi-omics toolbox of systems biology, targeted liquid chromatography-selected reaction monitoring (LC-SRM) measurements are critical for enzyme and pathway identification and design in metabolic engineering. To fulfill the increasing need for analyzing large sample sets with faster turnaround time in systems biology, high-throughput LC-SRM is greatly needed. Even though nanoflow LC-SRM has better sensitivity, it lacks the speed offered by microflow LC-SRM. Recent advancements in mass spectrometry instrumentation significantly enhance the scan speed and sensitivity of LC-SRM, thereby creating opportunities for applying the high speed of microflow LC-SRM without losing peptide multiplexing power or sacrificing sensitivity. Here, we studied the performance of microflow LC-SRM relative to nanoflow LC-SRM by monitoring 339 peptides representing 132 enzymes in *Pseudomonas putida* KT2440 grown on various carbon sources. The results from the two LC-SRM platforms are highly correlated. In addition, the response curve study of 248 peptides demonstrates that microflow LC-SRM has comparable sensitivity for the majority of detected peptides and better mass spectrometry signal and chromatography stability than nanoflow LC-SRM.

## Introduction

Liquid chromatography (LC) selected reaction monitoring (SRM, or multiple reaction monitoring – MRM) targeted proteomics is a popular mass spectrometry (MS)-based protein quantification technique (Picotti and Aebersold, 2012; Ebhardt et al., 2015). Highly sensitive and accurate protein quantification is afforded by LC-SRM analysis of enzymatic digests of proteins in the presence of isotope-labeled internal peptide standards. Most targeted proteomics assays are limited to a few dozen proteins per run and the samples are often acquired using nanoflow LC-SRM in order to achieve high sensitivity (Picotti et al., 2009; Huttenhain et al., 2012). As mass spectrometry techniques advance, the sensitivity and scan speed of mass spectrometers have greatly improved, yielding analyte detection with high signal to noise in short dwell times. LC-SRM workflows can now monitor hundreds of peptides in a single analysis (Lee et al., 2020), which provides researchers opportunities for deeper exploration into biological systems. In the current era of high-throughput biology, there is also an increasing need to systematically capture detailed information about biological systems with high-throughput experiments. Therefore, the long overhead time (i.e., sample loading, column washing, and equilibrating) of nanoflow LC-SRM can no longer meet the demands of high-throughput studies. Nanoflow LC-SRM also lacks robustness due to the difficulty in keeping stable electrospray over a long period of time. Compared to nanoflow LC-SRM, microflow LC-SRM provides higher throughput and better reproducibility, advantages that overshadow its slightly less sensitivity (Bian et al., 2020).

*Pseudomonas putida* KT2440 (*P. putida*) is a metabolically versatile, Gram-negative soil bacterium with excellent environmental tolerance since it can grow on a wide variety of carbon sources and thrive in diverse environmental conditions (e.g., aquatic and soil). It has considerable potential for a wide range of biotechnological applications (Linger et al., 2014; Loeschcke and Thies, 2015; Nikel et al., 2016; Johnson et al., 2019). It is critical to understand the intrinsic metabolism of *P. putida* before redesigning it to function as an efficient cell factory for desired bioproduct production through synthetic biology-guided engineering.

Here, we performed a systematic comparison of key characteristics of microflow LC-SRM and the conventional nanoflow LC-SRM platforms through a response curve study of 248 *P. putida* peptides in pooled *P. putida* digests, including throughput, sensitivity, reproducibility, and stability. We also applied both platforms to quantify the expression levels of 132 enzymes (i.e., 339 peptides) in *P. putida*, including enzymes from carbohydrate metabolism, amino acid metabolism, and other pathways. The bacteria were grown in 8 different conditions (*p*-coumarate in MOPS medium, glucose in MOPS medium, glucose in M9 medium, gluconate in M9 medium, fructose in M9 medium, glucose plus gluconate in M9 medium, fructose plus glucose in M9 medium, and fructose plus glucose plus gluconate in M9 medium). Together, we demonstrated that microflow LC-SRM is a robust, high-throughput targeted proteomic approach with little or no loss of sensitivity relative to nanoflow LC-SRM, and it works well in quantifying metabolic pathway enzymes and providing deep insights into the metabolism of *P. putida*.

## Materials and Methods

### *P. putida* KT2440 Cell Cultivation

*Pseudomonas putida* KT2440 cells were grown in either MOPS minimal media (LaBauve and Wargo, 2012) or modified minimal M9 medium (comprising 6.78 g/L Na_2_HPO_4_, 3 g/L KH_2_PO_4_, 0.5 g/L NaCl, 1 g/L NH_4_Cl, 2 mM MgSO_4_, 100 μM CaCl_2_, 18 μM FeSO_4_), and 30 mM total of the respective carbon source(s). In MOPS minimal media, cell cultures were cultivated in two individual carbon sources, glucose and *p*-coumarate. In modified minimal M9 medium, cell cultures were cultivated in three individual carbon sources, as well as permutations of each carbon source combination. Specifically, cell cultures were grown individually on glucose, gluconate, and fructose, and on mixed carbon sources: glucose plus gluconate, fructose plus glucose, fructose plus glucose plus gluconate. Cell cultures were inoculated to a starting optical density measured at 600 nm (OD_600_, measured by a Beckman DU640 spectrophotometer) of 0.1 in 50 mL of medium, according to previously reported methods (Bentley et al., 2020). The cultures were then incubated at 30°C in 250 mL baffled flasks, shaking at 225 rpm until an OD600 of 0.7 was reached, reflecting mid-log phase of growth. The cells were pelleted by centrifugation at 4,500 rpm for 5 min, the supernatant was decanted, and the pellets were washed with ice cold phosphate-buffered saline (PBS) and flash frozen in liquid nitrogen and stored at −80°C until analysis.

### Protein Extraction and Tryptic Digestion

Proteins were extracted from the cell pellets using a metabolite, protein, lipid extraction (MPLEx) method (Nakayasu et al., 2016; Burnum-Johnson et al., 2017; Kim and Heyman, 2018). Briefly, in solvent resistant tubes (Sorenson), the cell pellets were resuspended in H_2_O and a solvent mixture of four volumes of cold 2:1 chloroform:methanol mix was added. Samples were vigorously vortexed for 30 s, placed on ice for 5 min, vortexed again for 30 s, and centrifuged at 15,000 × *g* for 5 min at 4°C. After centrifugation, the denatured protein interphase was washed in 1 mL of cold 100% methanol, vortexed, and centrifuged again at 15,000 × *g* for 5 min at 4°C to pellet the protein. The methanol was removed, and samples were dried in a fume hood.

The protein pellet was resuspended in 100 mM NH_4_HCO_3_ containing 8 M urea and protein concentration was measured by a bicinchoninic acid (BCA) assay (Thermo Fisher Scientific, Waltham, MA, United States). Disulfide bonds were reduced by adding dithiothreitol (DTT) to a final concentration of 5 mM and incubating at 60°C for 30 min with constant shaking at 850 rpm. Samples were alkylated with a final concentration of 40 mM iodoacetamide for 1 h at 37°C at 850 rpm. The reaction was then diluted 10-fold with 100 mM NH_4_HCO_3_ followed by the addition of CaCl_2_ to 1 mM final concentration. Sequencing-grade trypsin (Promega, Madison, WI, United States) was added to all protein samples at a 1:50 (w/w) trypsin-to-protein ratio and incubated for 3 h at 37°C. Digested samples were desalted with 1 mL Discovery C18 SPE columns (Supelco, Bellefonte, PA, United States), using the following protocol: 3 mL of methanol was added for conditioning the column followed by 2 mL of 0.1% TFA in H_2_O. The samples were then loaded onto each column followed by 4 mL of 95:5: H_2_O:ACN, 0.1% TFA. Samples were eluted with 1 mL 80:20 ACN:H_2_O, 0.1% TFA. The samples were concentrated down to ∼100 μL using a Speed Vac and a final BCA was performed to determine the peptide concentration.

### Targeted Proteomics Assay Development

Targeted peptides were selected for 132 proteins in major pathways of *P. putida* KT2440, including carbohydrate metabolism, amino acid metabolism, biosynthesis of terpenoids and polyketides, energy metabolism, xenobiotics biodegradation, lipid metabolism, and nucleotide metabolism pathways (Supplementary Table 1) derived from RefSeq assembly accession GCF_000007565.2 using a BioCyc pathway/genome database (Caspi et al., 2013; Paley et al., 2017; Karp et al., 2019). Peptide selection was based on the spectral count data from our global proteomics and then evaluated by Prego (Searle et al., 2015) and CONSeQuence (Eyers et al., 2011) scores. All peptides were further blasted against *P. putida* KT2440 proteome using Protein Coverage Summarizer^1^ for their uniqueness to target proteins. The crude synthetic heavy isotope-labeled (e.g., ^13^C/^15^N on C-terminal lysine and arginine unless otherwise noted) peptides were purchased from New England Peptide (Gardner, MA, United States; FlashPure^TM^ Custom Peptide Array Tier 3). All the cysteines of the synthetic heavy peptides were modified by carbamidomethylation (CAM). Upon receiving the crude synthetic heavy peptides, they were mixed together and diluted with 0.1% formic acid in 15% acetonitrile in water to get a nominal concentration of 1 μM for each individual peptide. The heavy peptide stock solution was aliquoted and stored at −80°C until further use.

To evaluate the peptide quality and select the best responsive transitions for each peptide, 500 fmol/μL of heavy peptide mixture was subjected to high-resolution mass spectrometry analysis (e.g., LTQ Velos Orbitrap MS) since the peptide fragmentation patterns from HCD MS/MS on Orbitrap MS is similar to those from CID MS/MS on triple quadrupole MS (Wu et al., 2014). Firstly, the six most intensive fragment ions for each peptide were selected based on their corresponding MS/MS spectra. The collision energies for individual transitions were obtained by using empirical equations from the Skyline software (MacLean et al., 2010). Secondly, we employed LC-SRM to further evaluate all heavy peptides for the LC performance (e.g., the stability of peptide retention time), MS response (e.g., reliable heavy peptides identification), transition interferences, and endogenous peptide detectability by spiking them into water and the samples. In the end, 2–3 transitions per peptide and 1–3 peptides per protein were selected for the final panel of targeted proteomics assay. There were 339 peptides representing 132 proteins monitored in the final assay (Supplementary Tables 1, 2).

### Heavy Peptide Spike-In and Sample Loading

There are two sets of samples. One is individual *P. putida* samples taken directly from peptide digests, and the other is the pooled *P. putida* samples, where the individual samples were pooled together to make peptide digests with large volume and used exclusively for response curve studies.

For individual *P. putida* samples of microflow LC-SRM analysis, crude heavy peptide mixture stock solution was spiked in the 0.50 μg/μL peptide samples at a nominal concentration of 25 fmol/μL for each peptide. For individual *P. putida* sample of nanoflow LC-SRM analysis, crude heavy peptide mixture stock solution was spiked in the 0.125 μg/μL peptide samples at a nominal concentration of 6.25 fmol/μL for each peptide.

In the response curve study, the response curves of 248 peptides representing 111 proteins were evaluated by spiking heavy isotope labeled peptides in pooled *P. putida* samples at concentrations of 0 (blank), 0.002, 0.008, 0.04, 0.24, 1.2, 6, 30, 120, and 600 fmol/μg. Each of the above samples was subject to both microflow LC-SRM and nanoflow LC-SRM with loading of 25 μg for microflow LC-SRM and 0.25 μg for nanoflow LC-SRM. The response curve samples were injected from lowest to highest with triplicated technical replicates performed on each sample and platform combination.

### Microflow and Nanoflow LC-SRM Analysis

Microflow LC-SRM analysis utilized a nanoACQUITY H-Class UHPLC^®^ system (Waters Corporation, Milford, MA, United States), while nanoflow LC-SRM analysis utilized a M-Class UHPLC^®^ system (Waters Corporation, Milford, MA, United States). Both are coupled online to a TSQ Altis^TM^ triple quadrupole mass spectrometer (Thermo Fisher Scientific). The microflow LC-SRM’s UHPLC^®^ system was equipped with an ACQUITY UHPLC BEH 1.7 μm C18 column (1,000 μm i.d. × 15 cm), while the nanoflow LC-SRM’s UHPLC^®^ system was equipped with an ACQUITY UHPLC BEH 1.7 μm C18 column (100 μm i.d. × 10 cm). In both systems, the mobile phases were (A) 0.1% formic acid in water and (B) 0.1% formic acid in acetonitrile. The sample loading for microflow LC-SRM is 50 μL of sample (i.e., 25 μg of peptides), while that for nanoflow LC-SRM is 2 μL of sample (i.e., 0.25 μg of peptides). The gradient profile for the microflow LC contained a duty cycle of 32.0 min and a gradient length of 18.9 min (detailed as following, 0.0:90:7, 1.1:90:7, 12.0:90:28, 18.0:90:60, 20.0:90:95, 22.0:90:95, 23.0:90:1, 24.0:90:50, 25.0:90:1, 26.0:90:7, 32.0:90:7, in terms of min:flow-rate-μL/min:%B), while the gradient profile for the nanoflow LC contained a duty cycle of 110.0 min and a gradient length of 78.0 min (detailed as following, 0.0:0.4:1, 1.0:0.6:1, 6.0:0.6:1, 7.0:0.4:1, 9.0:0.4:6, 40.0:0.4:13, 70.0:0.4:22, 80.0:0.4:40, 85.0:0.4:95, 90.0:0.4:95, 91.0:0.5:95, 92.0:0.5:95, 93.0:0.5:50, 94.0:0.5:95, 95.0:0.6:1, 98.0:0.4:1, 110.0:0.4:1, in terms of min:flow-rate-μL/min:%B). Both LC columns were operated with a temperature of 45°C. The TSQ Altis^TM^ triple quadrupole mass spectrometer was operated with ion spray voltages of 4000 ± 100 V and a capillary inlet temperature of 325°C in microflow SRM mode, while it was operated with ion spray voltages of 2100 ± 100 V and a capillary inlet temperature of 350°C in nanoflow SRM mode. In both microflow LC-SRM and nanoflow LC-SRM, tube lens voltages were obtained from automatic tuning and calibration without further optimization. Both Q1 and Q3 were set at unit resolution of 0.7 FWHM and Q2 gas pressure was optimized at 1.5 mTorr. The transitions were scanned with a minimal dwell time of 0.879 msec for microflow SRM and 0.806 msec for nanoflow SRM, respectively.

### Data Analysis

All the LC-SRM data were imported into the Skyline software and the peak boundaries were manually inspected to ensure correct peak assignment and peak boundaries. The normalized dot product of the light transition peak areas with the heavy transition peak areas (i.e., DotProduct as denoted in Skyline) was calculated by the Skyline software, and it can be used to check whether the transition peak areas in the two label types are in the same ratio to each other determining their spectral similarity.

For individual samples, the total peak area ratios of endogenous light peptides and their corresponding heavy isotope-labeled internal standards were calculated by the Skyline software. The detectability of a spectra was defined by having DotProduct above 0.86 and maximum intensity of light above 1,300. Peptide-peptide correlation within single proteins were evaluated and there were 16 peptides whose abundance profile across 30 samples were significantly different from other peptides in the same proteins. The final 323 peptides were proceeded with final protein abundance rollup by removing those 16 peptides. Specifically, data were log2 transformed, compared to assure no biases (Webb-Robertson et al., 2011), and normalized by global median centering based on rank-invariant peptides (Callister et al., 2006), where rank invariance was determined by a *p*-value threshold of 0.2. Protein quantification was performed using R-rollup (Polpitiya et al., 2008; Matzke et al., 2013), which scaled the peptides associated with each protein by a reference peptide (the peptide with the least missing data) and then set the median of the scaled peptides as the protein abundance. Pairwise-univariate statistical comparisons were carried out between each of the other carbon sources and glucose in M9 medium using an analysis of variance (ANOVA) with a Dunnet multiple test correction, or between *p*-coumarate and glucose in MOPS medium and between MOPS medium and M9 medium with glucose as carbon source using a standard two-sample *t*-test (Webb-Robertson et al., 2010, 2017).

For response curve study, the response curves of peptides were generated using the heavy-over-light peak area ratios and the heavy peptides concentrations. Similar to the analysis of individual samples, the DotProduct for all the replicates at the LODs and LOQs level need to be above 0.86 while the maximum intensity of heavy above 1,300. The limit of detection (LOD) was determined from the blanks using the average plus three times the standard deviation of the blank signals, while the limit of quantification (LOQ) using the average plus 10 times the standard deviation of the blank signals. Additionally, LOQs also have coefficient variations of less than 20%. The final LOD and LOQ were listed in Supplementary Table 3. Peak capacity was calculated using the formula p = 1 + t_*g*_/w, where t_*g*_ is the length of the length of the LC gradient and w is the peak width in terms of the full width at half maximum (FWHM). FWHMs were exported from Skyline. The gradient lengths are 78.0 min and 18.9 min for nanoflow and microflow LC-SRM, respectively, while the average FWHMs of all the 248 response curve peptides are 0.22 min and 0.12 min for nanoflow and microflow LC-SRM, respectively. The calculated peak capacities are 356 and 159 for nanoflow and microflow LC-SRM, respectively.

## Results

**FIGURE 1:**
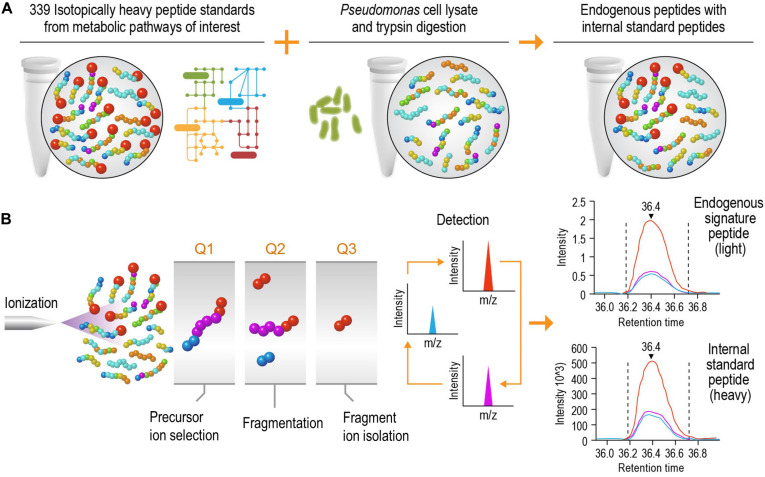
Schematic diagram of the liquid chromatography selected reaction monitoring (LC-SRM) workflow. **(A)** Sample establishment. The heavy isotope labeled internal standard peptides from metabolic pathways of interest are spiked into the tryptic peptides digested from cell lysates, and this results in a mixture of endogenous peptides and internal standard peptides. The mixture will go through liquid chromatography separation and be analyzed by mass spectrometers. **(B)** Selected reaction monitoring using a triple quadrupole mass spectrometer. The eluents of liquid chromatography (LC) separation, carrying both endogenous and internal standard peptides at the same time, are ionized using electrospray ionization. The precursor ions are filtered in Q1, and they are then dissociated via collision into multiple fragment ions in Q2. In Q3, each fragment ion is monitored individually (normally 1 to 5 fragment ions per precursor ion) over the LC elution time. The endogenous peptides share the same retention time and fragmentation profile as their internal standard counterparts. The authors thank PNNL Graphic Designer Nathan Johnson for preparing the figure.

The goal of this study was to develop a high-speed platform that can expedite targeted proteomics analysis, thereby increasing the sample analysis throughput for studying biological systems without significantly reducing the sensitivity. Implementation of this high-speed LC-SRM platform can analyze enzymatic digests of *P. putida* protein extracts in the presence of hundreds of isotope-labeled internal peptide standards, enabling rapid and accurate protein quantification and deep exploration of metabolic pathways (Figure 1).

### Microflow and Nanoflow LC-SRM Platform Comparison in Response Curve Study

**FIGURE 2:**
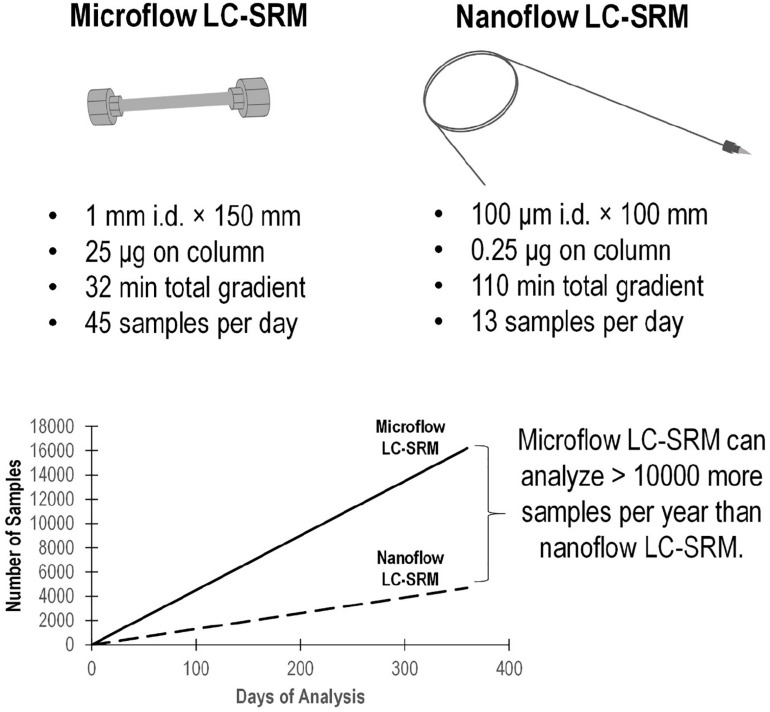
Comparison of the microflow LC-SRM and nanoflow LC-SRM platforms. The top figure includes the size of the column, sample loading, total gradient length, and number of samples per day. The bottom figure shows the number of samples run versus the days spent on the analysis in an ideal situation regardless of the instrument down time. In 1 year, the microflow LC-SRM platform can analyze >10000 more samples than the nanoflow LC-SRM platform.

The microflow LC-SRM platform utilized a 1 mm i.d. column packed with 1.7 μm C18 particles, with a total run time of 32 min. By comparison, the nanoflow LC-SRM system employed a 100 μm i.d. column packed with the same C18 particles, with a total run time of 110 min, and thus the microflow LC-SRM platform increases the sample analysis throughput by more than 3-fold. The microflow LC-SRM platform can potentially result in analyzing 10,000 more samples than the nanoflow LC-SRM system each year (Figure 2). Many software tools (MacLean et al., 2010; Choi et al., 2014; Gibbons et al., 2019) can be used to facilitate the efficient analysis of the large-scale SRM data, and the data analysis time will be well below the instrument run time. The utilization of microflow LC-SRM is often considered to have lower sensitivity but increased robustness relative to the nanoflow LC-SRM system (Gatlin et al., 1998; Bian et al., 2020). In order to evaluate the effectiveness and efficacy of microflow LC-SRM, we performed a thorough comparison between microflow LC-SRM and nanoflow LC-SRM.

**FIGURE 3:**
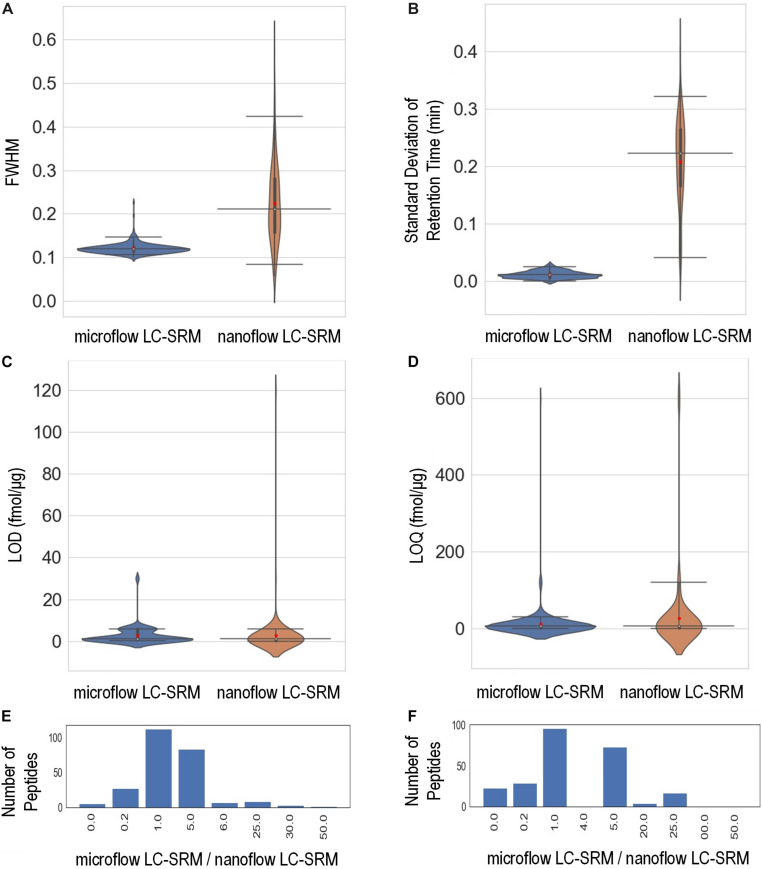
Performance characteristics of the microflow LC-SRM system versus nanoflow LC-SRM system from the response curve study of 248 *P. putida* peptides. The microflow LC-SRM platform is shown in blue and the nanoflow LC-SRM platform in brown for **(A–D)**. **(A)** Violin plot comparing the average full width at half maximum (FWHM) of the three replicated samples at 600 fmol/μg heavy isotope labeled peptide spike-in concentration. **(B)** Violin plot comparing the standard deviation of the retention times of the three replicated samples at 600 fmol/μg heavy isotope labeled peptide spike-in concentration. **(C)** Violin plot comparing the limit of detection (LOD). **(D)** Violin plot comparing the limit of quantification (LOQ). **(E)** Histogram of the LOD differences between microflow LC-SRM platform and nanoflow LC-SRM platform of individual peptides, in terms of ratios of LOD in microflow LC-SRM over LOD in nanoflow LC-SRM. **(F)** Histogram of the LOQ difference between the microflow LC-SRM platform and nanoflow LC-SRM platform of individual peptides, in terms of ratios of LOQ in microflow LC-SRM over LOQ in nanoflow LC-SRM. The three horizontal lines across the violin plots are 2.5, 50, and 97.5% quartiles, respectively, while the red dots in the violin plots are the mean value.

The study monitored the responses of 248 heavy isotope labeled synthetic peptides (Supplementary Table 3) spiked-in at various concentrations on both microflow LC-SRM and nanoflow LC-SRM platforms. The endogenous peptides in the pooled *P. putida* samples were used here as references in LC-SRM analysis. The effective gradient length of nanoflow LC-SRM is about five times that of microflow LC-SRM (Supplementary Figure 1). On average, the chromatographic peak width (FWHM) in microflow LC-SRM is about 2 times that of nanoflow LC-SRM (Figure 3A). The resulting peak capacity of the nanoflow LC-SRM is substantially higher than that of the microflow LC-SRM (>2 times). This indicates that the extended LC gradient greatly improves peak capacity, even when the column length of microflow LC-SRM (150 mm) is longer than that of nanoflow LC-SRM (100 mm). The lower peak capacity of microflow LC-SRM resulted in lower separation power compared to that of nanoflow LC-SRM, as seen in their total ion chromatography (Supplementary Figure 1). Even though microflow LC-SRM will benefit from the narrower peak width by increasing analyte concentration, the high flow significantly dilutes analyte concentration (90 μL/min versus 0.4 μL/min). To offset this dilution, 100 times more sample mass was loaded on to the microflow LC-SRM column. Sample loading might be a concern for some biological studies with limited sample volumes. By loading 100 times more sample onto the column, similar concentrations of analytes at the time of elution were achieved between nanoflow LC-SRM and microflow LC-SRM. The peak areas of peptides in nanoflow LC-SRM were on average 4 times higher than those in microflow LC-SRM (Supplementary Figures 2A,C), mainly due to less interference and better ionization efficiency of nanoflow LC-SRM. However, the stability of the ESI signal in microflow LC-SRM is much higher than that in nanoflow LC-SRM, as demonstrated by the coefficient of variation (CV) of peptide peak areas of three replicated samples (Supplementary Figures 2B,D) as well as the CV of peptide peak area ratios of three replicated samples (Supplementary Figure 2E). Moreover, the peptide retention time is also more stable in microflow LC-SRM compared to that in nanoflow LC-SRM. As shown in Figure 3B, the average standard deviation of peptide retention time is 0.21 min for nanoflow LC-SRM while 0.01 min for microflow LC-SRM. The standard deviation of peptide retention time is an important factor in determining the time window (i.e., start and end times of acquisition) of each peptide for a large-scale multiplexed LC-SRM assay. The smaller the standard deviation of peptide retention time, the narrower the time window. Microflow LC-SRM will be able to use a much narrower time window to fit more peptides in the assay, which will improve the peptide-multiplexing power in a single LC-SRM run.

In order to evaluate the sensitivity of microflow LC-SRM and nanoflow LC-SRM, the LODs and LOQs of 248 peptides were measured. The LODs and LOQs of all the 248 peptides are listed in Supplementary Table 3. In general, the distributions of overall LODs and LOQs in microflow LC-SRM are very similar to those in nanoflow LC-SRM (Figures 3C,D). Comparing the LODs and LOQs of microflow LC-SRM to those of nanoflow LC-SRM at the individual peptide level (Figures 3E,F), the LODs and LOQs of the majority of peptides are the same, while the number of peptides whose LODs are higher in microflow LC-SRM are more than the number of peptides whose LODs are lower in microflow LC-SRM. This indicates that microflow LC-SRM provides equal or slightly lower sensitivity compared to nanoflow LC-SRM. Microflow LC-SRM is likely to lose some sensitivity compared to nanoflow LC-SRM due to its smaller peak area and lower separation power, but the increased stability of microflow LC-SRM overcomes this potential limitation.

In summary, compared to nanoflow LC-SRM, microflow LC-SRM has comparable or slightly lower sensitivity and similar multiplexing power, but much better sample throughput and stability. The main criteria for applying microflow LC-SRM is whether there is enough biological material (i.e., 25–50 μg of peptide digests) to load onto the larger microflow analytical column.

### LC-SRM Analysis of Metabolic Pathway Enzymes in 30 *P. putida* Samples

Both microflow LC-SRM and nanoflow LC-SRM were used to analyze a total of 132 enzymes, including 92 in carbohydrate metabolism, 26 in amino acid metabolism, 4 in nucleotide metabolism, 3 in energy metabolism, 4 in biosynthesis of terpenoids and polyketides, 2 in lipid metabolism, and 1 in xenobiotics biodegradation, as listed in Supplementary Table 1. All the 339 peptides corresponding to 132 proteins monitored were detected by both microflow LC-SRM and nanoflow LC-SRM. The detectability (i.e., number of samples where peptides are detected) in the majority of the peptides (i.e., 304 peptides) are the same between microflow LC-SRM and nanoflow LC-SRM, while nanoflow LC-SRM has slightly better detectability in 34 peptides and worse detectability in 1 peptide than microflow LC-SRM (see Supplementary Table 4). Overall, the peptide abundance measured by microflow LC-SRM and nanoflow LC-SRM are highly correlated for the same sample (Supplementary Figure 3).

In this study, the samples fed with glucose were grown in either M9 minimal salts medium or MOPS minimal medium, while samples fed with *p*-coumarate were grown in MOPS medium and samples fed with gluconate, fructose and mixed carbon sources (glucose + gluconate, fructose + glucose, fructose + glucose + gluconate) were grown in M9 medium. Statistical comparisons between conditions at protein level demonstrated great similarity between microflow LC-SRM and nanoflow LC-SRM in their findings of significantly differentiated proteins (i.e., *p*-value < 0.05 and fold change > 2), as shown in Supplementary Figures 4A–G.

The stability of peptide retention time across 30 samples in nanoflow LC-SRM is worse than that in microflow LC-SRM (Supplementary Figure 5), and these differences were larger when analyzing 30 different samples compared to only analyzing 3 samples of the same matrix composition in the response curve study (Figure 3B). The less stable peptide retention time using nanoflow LC-SRM will make the time window scheduling challenging, especially for the analysis of complex sample extracts. Microflow LC-SRM is better suited to facilitate high-throughput time-scheduled SRM transition acquisition of large target numbers (hundreds of peptides) across large sample sets.

### Enzyme Expression Levels of *P. putida* KT2440 Strains Grown in Different Carbon Sources

Central carbon metabolism (Supplementary Figure 6) consists of a series of enzymatic activities to convert carbon sources into valuable metabolic precursors (Noor et al., 2010), and in *P. putida* includes the Embden-Meyerhof-Parnas (EMP) pathway of glycolysis, pentose phosphate (PP) pathway, Entner-Doudoroff (ED) pathway, anaplerosis routes, and tricarboxylic acid cycle (TCA) (Nikel et al., 2015). *P. putida* can grow on a wide variety of carbon sources, from multiple carbohydrates (e.g., glucose, gluconate, fructose) to aromatic carbon (e.g., *p*-coumarate). Glucose and gluconate are transported into the cell either directly or through the conversion process of glucose to gluconate to 2-ketogluconate in the periplasmic space (Supplementary Figure 6) (Rojo, 2010). Once inside, glucose, gluconate, and 2-ketogluconate go through the initial glucose catabolism pathways and converge onto the central carbon metabolism (Chavarria et al., 2012). In contrast, fructose is transported by phosphoenolpyruvate-dependent sugar phosphotransferase system (PTS) and converted to fructose-1,6-bisphosphate by 1-phosphofructokinase encoded by genes in the fruBKA operon (Chavarria et al., 2016). *P. putida* lacks a classical EMP pathway due to the absence of 6-phosphofructokinase, and utilizes these hexose sugars through a cycle formed by enzymes in the ED, EMP, and PP pathways (Nikel et al., 2015). On the other hand, *p*-coumarate is metabolized via the β-ketoadipate pathway before joining the central carbon metabolism via acetyl-CoA and succinate.

Among the 132 enzymes monitored in this study, 83 of them comprise central carbon metabolism, the β-ketoadipate pathway, and the initial glucose catabolism pathways, as shown in Supplementary Figure 6. Overall, microflow LC-SRM and nanoflow LC-SRM resulted in similar quantitative patterns across 84 proteins and 30 biological samples and the hierarchy of clustering of genes obtained after performing unsupervised clustering was the same for both platforms (Supplementary Figure 7).

**FIGURE 4:**
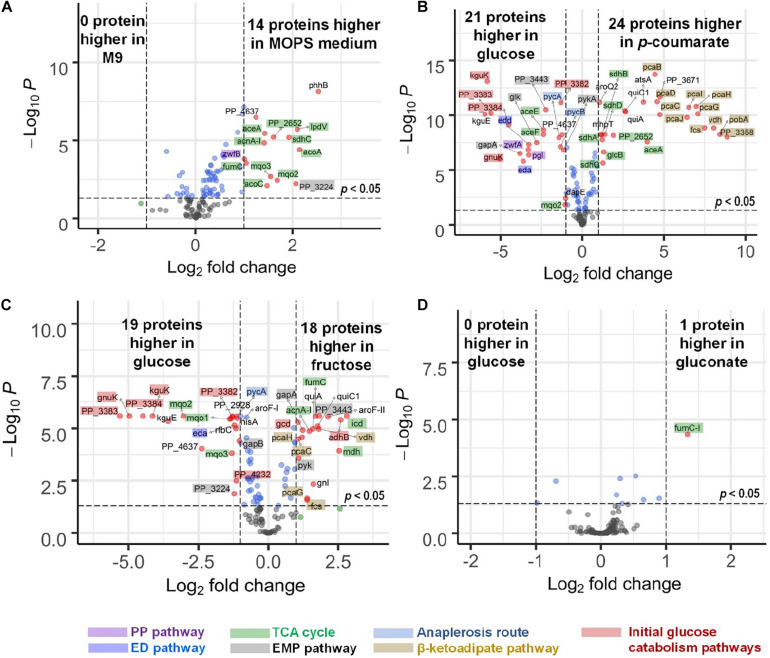
Volcano plots displaying differential expressed genes in four comparisons presented by microflow LC-SRM results of protein expression level of pathway genes in *P. putida*. **(A)** Comparison of strains grown in MOPS medium to those grown in M9 medium, both with glucose as carbon source. **(B)** Comparison of strains grown in *p*-coumarate against those grown in glucose, both in MOPS medium. **(C)** Comparison of strains grown in fructose against those grown in glucose, both in M9 medium. **(D)** Comparison of strains grown in gluconate against those grown in glucose, both in M9 medium. The vertical axis (*y*-axis) corresponds to the significance in terms of -log10 *P* (*p*-value), and the horizontal axis (*x*-axis) displays the log2 fold change value. The red dots represent significantly differentially expressed genes (*p*-value < 0.05, | fold change| > 2) that are either increased (right) or decreased (left); the blue dots represent the genes whose fold change is less than two folds in either direction but with enough significance (*p*-value < 0.05); the green dots represent genes whose fold change is more than two folds in either direction without enough significance (*p*-value > 0.05); the black dots represent genes whose fold change is less than two folds in either direction without enough significance (*p*-value > 0.05). All the significantly differentially expressed genes are label with their gene names. The total of variables plotted contain results of 132 genes. The shades on the gene labels indicate their pathway categories, including the Embden-Meyerhof-Parnas (EMP) pathway of glycolysis, pentose phosphate (PP) pathway, Entner-Doudoroff (ED) pathway, anaplerosis routes, tricarboxylic acid cycle (TCA), the initial glucose catabolism pathways, and β-ketoadipate pathway.

**FIGURE 5:**
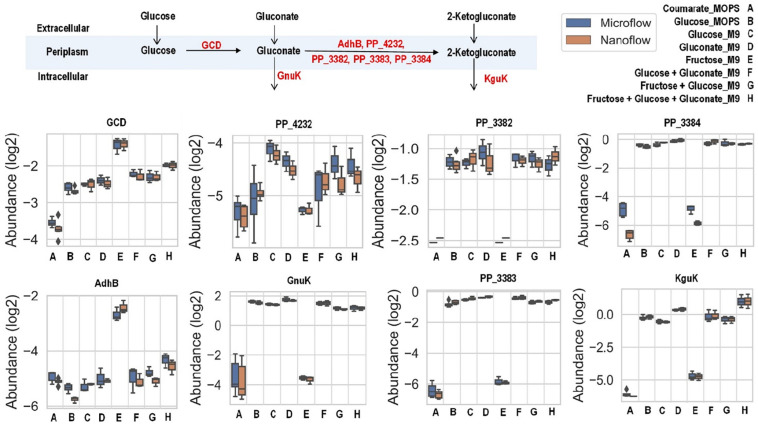
Boxplot of the relative abundance of enzymes in the initial glucose catabolism pathways of up-taking glucose and gluconate with *P. putida* grown on eight different growth conditions: A, *p*-coumarate in MOPS medium; B, glucose in MOPS medium; C, glucose in M9 medium; D, gluconate in M9 medium; E, fructose in M9 medium; F, glucose and gluconate in M9 medium; G, fructose and glucose in M9 medium; H, fructose, glucose and gluconate in M9 medium. The enzymes are quinoprotein glucose dehydrogenase (GCD), alcohol dehydrogenase cytochrome c subunit (AdhB), cytochrome c family protein (PP_4232), gluconate 2-dehydrogenase cytochrome c subunit (PP_3382), gluconate 2-dehydrogenase flavoprotein subunit (PP_3383), gluconate 2-dehydrogenase gamma subunit (PP_3384), gluconokinase (GnuK), and putative 2-ketogluconokinase (KguK). Each box represents the distribution of expression levels of the corresponding enzymes in at least three independent biological replicated samples, including minimum (bottom bar), maximum (top bar), median (line inside the box), first quartile (bottom edge of the box), third quartile (top edge of the box) and diamond (outliers).

There are slight differences of enzyme expression levels between glucose-fed samples grown in MOPS medium versus those in M9 medium, and the major variant enzymes between the two conditions are the ones in the TCA, EMP, and PP pathways (see Figure 4A). Comparing strains grown on different carbon sources in the same medium (for example, *p*-coumarate versus glucose in MOPS medium, fructose versus glucose in M9 medium), the enzymes in pathways associated with the intracellular entering route of the carbon sources into *P. putida* have the most significantly (i.e., *p*-value < 0.05 and fold change > 2) altered expression levels. When *p*-coumarate is the sole carbon source, the majority of the enzymes in the β-ketoadipate pathway are increased, while the majority of the enzymes in the initial glucose catabolism pathways and all the enzymes in the ED pathway are significantly decreased (Figure 4B). However, few enzymes in the EMP pathway, PP pathway, and TCA cycle are significantly altered, while the enzymes in the TCA cycle exhibit some differences. Namely, some are decreased (AceE, AceF, and Mqo2) and others are increased (SdhA, SdhB, SdhC, SdhD, AceA, and PP_2652). When fructose is the sole carbon source, the downstream enzymes (i.e., GnuK, PP_4232, PP_3382, PP_3383, PP_3384, and KguK) in the initial glucose catabolism pathways of the glucose-gluconate uptake system are expressed at a very low level, but in contrast, the levels of early pathway enzymes (i.e., GCD and AdhB) in the periplasmic space are expressed at a significantly higher level compared to the presence of other carbon sources (Figures 4C, 5). In addition, several enzymes in the β-ketoadipate pathway are significantly increased and half of the enzymes in the ED pathway are significantly decreased (Figure 4C). However, only a few enzymes in the EMP pathway and TCA cycle are significantly altered and the rest show minor changes in relative abundance, and similar to what was observed in the comparison between *p*-coumarate and glucose, the enzymes in the TCA can be either decreased or increased depending on carbon sources.

Most of the enzymes are not altered significantly when comparing fructose mixed with either glucose or glucose plus gluconate against glucose (Supplementary Figures 4D,E), and gluconate either alone (Figure 4D) or mixed with glucose against glucose (Supplementary Figure 4G). The expression levels of only a few enzymes are changed significantly. This is likely due to either the convergence of the metabolic pathways utilized by gluconate and glucose and/or the co-presence of glucose in the system.

The uptake of glucose and gluconate into the cell are incorporated through the initial glucose catabolism pathways. Glucose can be converted to gluconate in the periplasmic space by quinoprotein glucose dehydrogenase (encoded by the *gcd* gene). Once in cytoplasm, glucose will first be phosphorylated by glucokinase (encoded by the *glk* gene) and then converted to 6-phosphogluconate by glucose-6-phosphate 1-dehydrogenase (encoded by the *zwfA*, *zwfB*, and *zwf* genes) followed with 6-phosphogluconolactonase (encoded by the *pgl* gene), while gluconate is phosphorylated directly to 6-phosphogluconate by gluconokinase (encoded by the *gnuK* gene). Interestingly, even grown solely in either glucose or gluconate (both with M9 medium), there are no variations of expression levels for the enzymes converting these carbon sources to 6-phosphogluconate, except slight increase of KguK in samples grown in gluconate (Figure 5).

**FIGURE 6:**
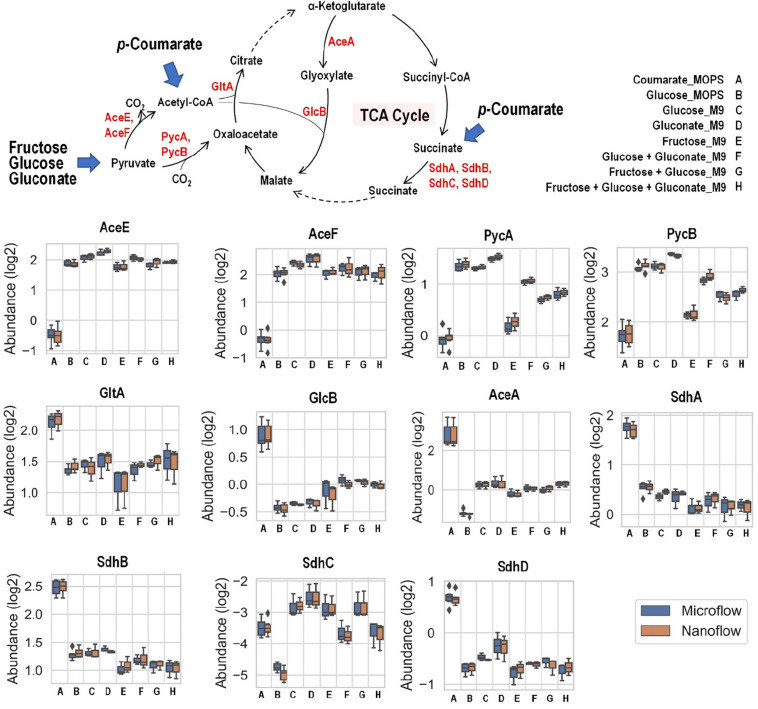
Boxplot of the relative abundance of enzymes in *P. putida* grown on eight different growth conditions: A, *p*-coumarate in MOPS medium; B, glucose in MOPS medium; C, glucose in M9 medium; D, gluconate in M9 medium; E, fructose in M9 medium; F, glucose and gluconate in M9 medium; G, fructose and glucose in M9 medium; H, fructose, glucose and gluconate in M9 medium. These enzymes either facilitate the entrance of key organic carbon products (pyruvate and acetyl-CoA) into the tricarboxylic acid cycle (TCA cycle) or fuel TCA cycle via succinate as substrates. While pyruvate is generated from aliphatic carbon sources (i.e., fructose, glucose and gluconate), acetyl-CoA and succinate are resulted from aromatic carbon source (i.e., *p*-coumarate). The enzymes are pyruvate carboxylase subunit A (PycA), pyruvate carboxylase subunit A (PycB), citrate synthase (GltA), malate synthase G (GlcB), isocitrate lyase (AceA), succinate dehydrogenase flavoprotein subunit (SdhA), succinate dehydrogenase iron-sulfur subunit (SdhB), succinate dehydrogenase membrane b-556 subunit (SdhC), and succinate dehydrogenase hydrophobic membrane anchor subunit (SdhD). Each box represents the distribution of expression levels of the corresponding enzymes in at least three independent biological replicated samples, including minimum (bottom bar), maximum (top bar), median (line inside the box), first quartile (bottom edge of the box), third quartile (top edge of the box) and diamond (outliers).

Fructose, glucose, and gluconate metabolism eventually converge to pyruvate and then into the TCA cycle, either directly or through acetyl-CoA as intermediate, while carbon from *p*-coumarate enters the TCA cycle through acetyl-CoA and succinate (Figure 6). When *p*-coumarate is the sole carbon source, the levels of enzymes at the entrance point of acetyl-CoA into TCA cycle (GltA and GlcB) and those utilizing succinate into TCA cycle (SdhA, SdhB, and SdhD) are relatively increased. In contrast, the levels of pyruvate carboxylase subunit A and B (PycA and PycB) and pyruvate dehydrogenase E1 and E2 component (AceE and AceF) are decreased when *p*-coumarate is used relative to glucose. This agrees with the fact metabolism of *p*-coumarate generates succinate and acetyl-CoA via β-ketoadipate without pyruvate. In *P. putida* KT2440, benzoate is also known to be degraded to succinate and acetyl-CoA via β-ketoadipate, and its catabolism has been well studied using kinetic modeling (Sudarsan et al., 2016), transcriptomics (Sudarsan et al., 2014), global proteomics and fluxomics (Kukurugya et al., 2019). The transcriptomics study found that these genes involved in pyruvate metabolism and TCA cycle were not differentially expressed at steady state between benzoate and glucose, but the downregulation of succinate dehydrogenase was observed transiently when carbon source was shifted from benzoate to glucose (Sudarsan et al., 2014). More similar observations were made in the proteomics study comparing cells grown on glucose and benzoate to glucose only, including the upregulation of citrate synthase and succinate dehydrogenase as well as the downregulation of pyruvate dehydrogenase (Kukurugya et al., 2019). Interestingly, different observation was made for the expression of genes involved in glyoxylate cycle. In the global proteomics study isocitrate lyase (AceA) was significantly downregulated comparing cells grown on glucose and benzoate to glucose only and malate synthase G (GlcB) was not detected in either cases (Kukurugya et al., 2019), whereas in our targeted proteomics study AceA and GlcB were both increased in *p*-coumarate versus glucose (Figure 6).

## Conclusion

In this study, we systematically compared the performance of two LC-SRM platforms, microflow LC-SRM and nanoflow LC-SRM, through monitoring hundreds of targeted peptides in response curve samples as well as individual samples grown in different environmental conditions. The results of this evaluation clearly demonstrated the promise of microflow LC-SRM as a robust protein quantification system biology tool with high sensitivity, high peptide-multiplexing capability, and high sample throughput. Compared to nanoflow LC-SRM, microflow LC-SRM improves the speed by 3-fold, while providing comparable sensitivity over hundreds of peptides. The results of 132 enzymes in *P. putida* reveals reliable and highly correlated quantification by microflow LC-SRM and nanoflow LC-SRM. In addition, the quantification of enzymes in the central carbon metabolism, the initial glucose catabolism pathways, and β-ketoadipate pathway reveals the changes of these enzyme expression levels of *P. putida* in response to various carbon sources and media composition. The increased throughput and measurement reliability of the presented microflow LC-SRM platform makes it an exceptional test tool for synthetic biology-guided engineering by reducing the cycle time of Design-Build-Test-Learn cycles for enhanced microbial bioproduct production.

## Data Availability Statement

The datasets presented in this study can be found in online repositories. The name of the repository and link can be found below: Panorama Public, https:// panoramaweb.org/ABF_P_Putida_KT2440_HighFlow_SRM.url.

## Author Contributions

KB-J and YG planned and designed the targeted proteomic studies. GTB, JG, AG, GJB, CJ, JAM, JKM, and KB-J planned and designed the *P. putida* studies. NM and MB prepared the samples for proteomic analysis. YG and TF performed the targeted proteomic experiments and data analysis. B-JW-R performed the statistical analysis. YG and JK performed the data analyses. YG wrote the first draft of the manuscript. All authors contributed to the revision of the manuscript.

## Conflict of Interest

The authors declare that the research was conducted in the absence of any commercial or financial relationships that could be construed as a potential conflict of interest. The reviewer LB declared a past co-authorship with one of the authors GTB to the handling editor.

## Footnotes

1 https://omics.pnl.gov/software/protein-coverage-summarizer
