# Supplementary material for: High-Throughput Large-Scale Targeted Proteomics Assays for Quantifying Pathway Proteins in Pseudomonas putida KT2440
Source: Front Bioeng Biotechnol. 2020 Dec 2;8:603488. doi: 10.3389/fbioe.2020.603488 (PMC7793925; doi:10.3389/fbioe.2020.603488)
Supplement: Supplementary file 1 [file Data_Sheet_1.docx]

Supplementary Figures


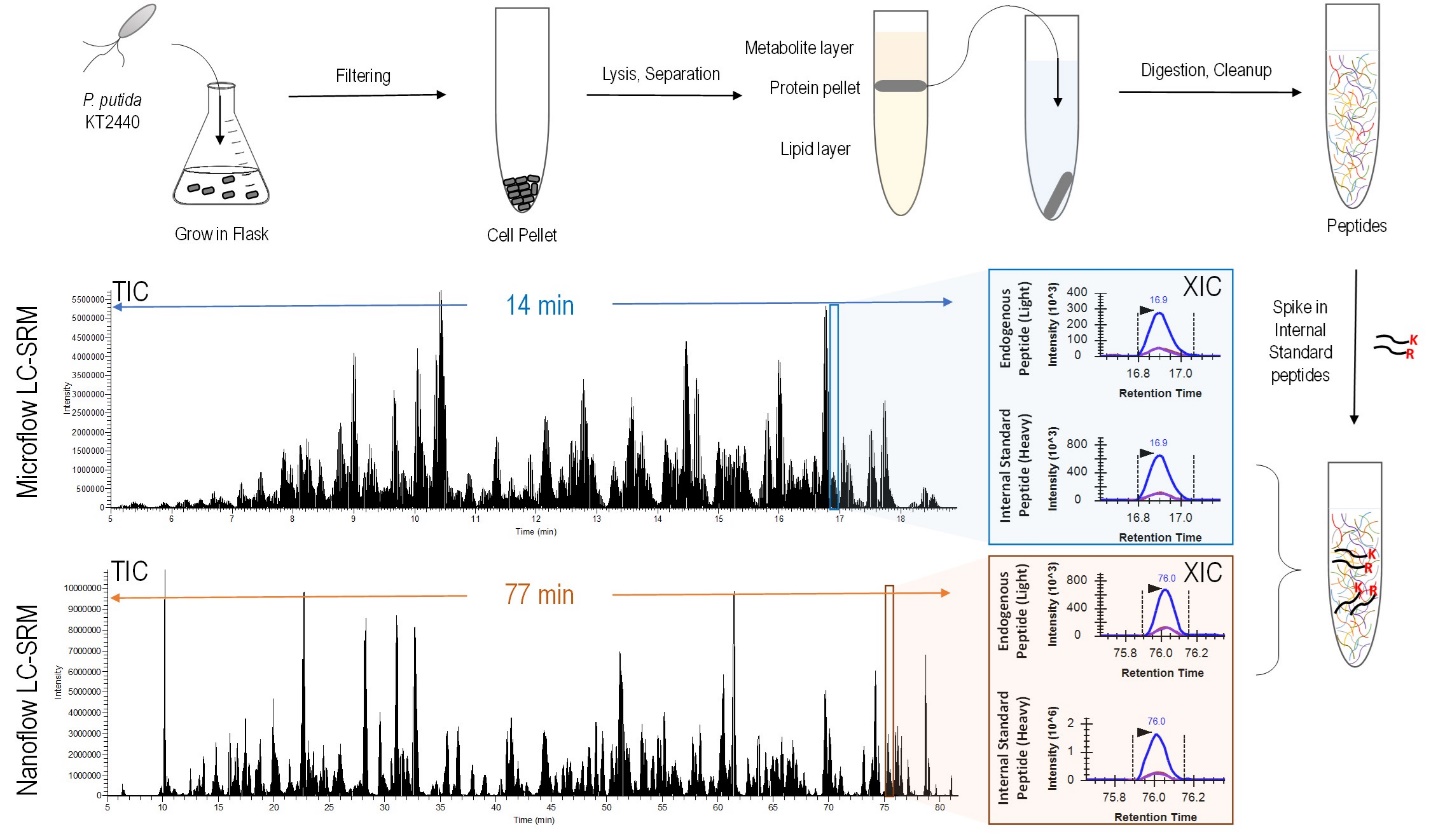


Supplementary Figure 1. Targeted proteomics workflow of *P. putida* KT2440 samples and the example extracted ion chromatography (XIC) and total ion chromatography (TIC) of microflow LC-SRM and nanoflow LC-SRM. The effective gradient length is 14 min for microflow LC-SRM and 77 min for nanoflow LC-SRM.


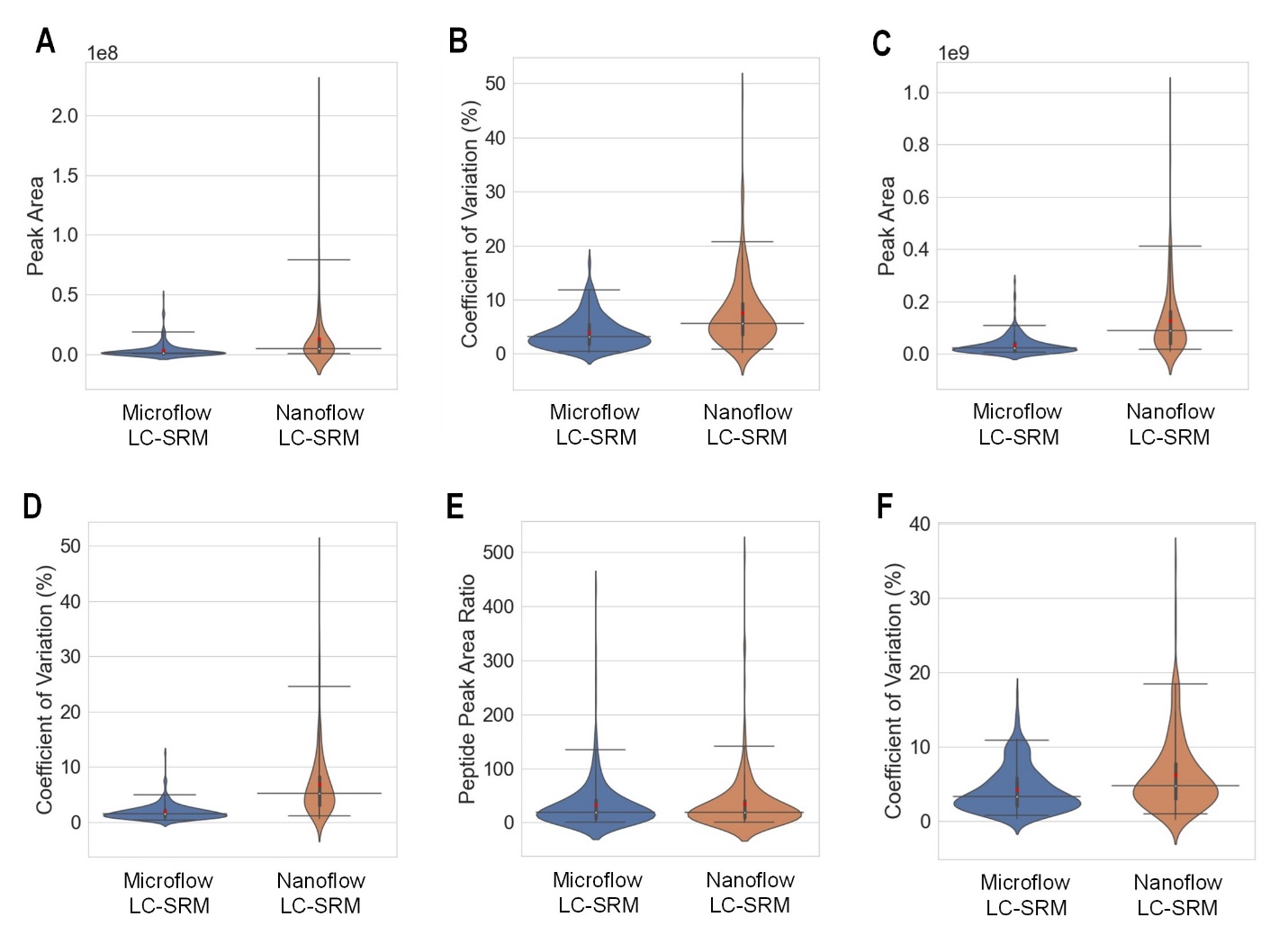


Supplementary Figure 2. Performance characteristics of the microflow LC-SRM system versus nanoflow LC-SRM system from the response curve study of 248 *P. putida* peptides. The microflow LC-SRM platform is shown in blue and the nanoflow LC-SRM platform in brown. A) Violin plot comparing the peak area of endogenous peptides. B) Violin plot comparing the coefficient of variation (CV) of the three peak areas of endogenous peptides in three replicated samples. C) Violin plot comparing the peak area of heavy isotope labelled peptides. D) Violin plot comparing the coefficient of variation (CV) of the three peak areas of heavy isotope labelled peptides in three replicated samples. E) Violin plot comparing peak area ratio. F) Violin plot comparing the coefficient of variation (CV) of the three peak area ratios in three replicated samples. The data was obtained from the three replicated samples at 600 fmol/μg heavy isotope labelled peptide spike-in concentration. Note: The three horizontal lines across the violin plots are 2.5%, 50% and 97.5% quartiles, respectively, while the red dots in the violin plots are the mean value.


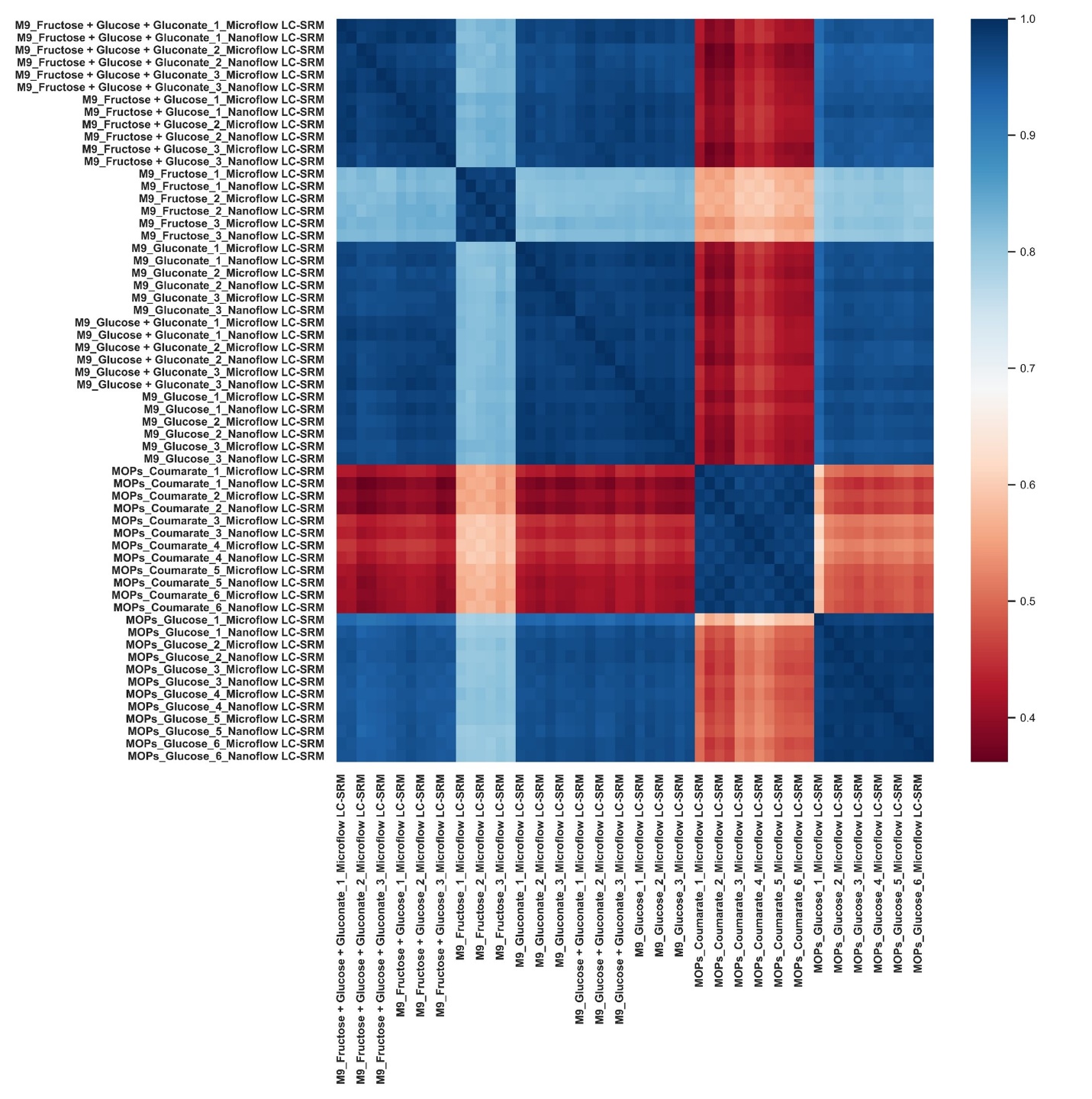


Supplementary Figure 3. Correlation plot between 30 biological samples using the peptide peak area ratio measured from both microflow LC-SRM and nanoflow LC-SRM, including 6 biological replicates of p-coumarate in MOPS medium, 6 biological replicates of glucose in MOPS mediu

m, 3 biological replicates of glucose in M9 medium, 3 biological replicates of gluconate in M9 medium, 3 biological replicates of fructose in M9 medium, 3 biological replicates of glucose and gluconate in M9 medium, 3 biological replicates of fructose and glucose in M9 medium, and 3 biological replicates of fructose, glucose and gluconate in M9 medium.


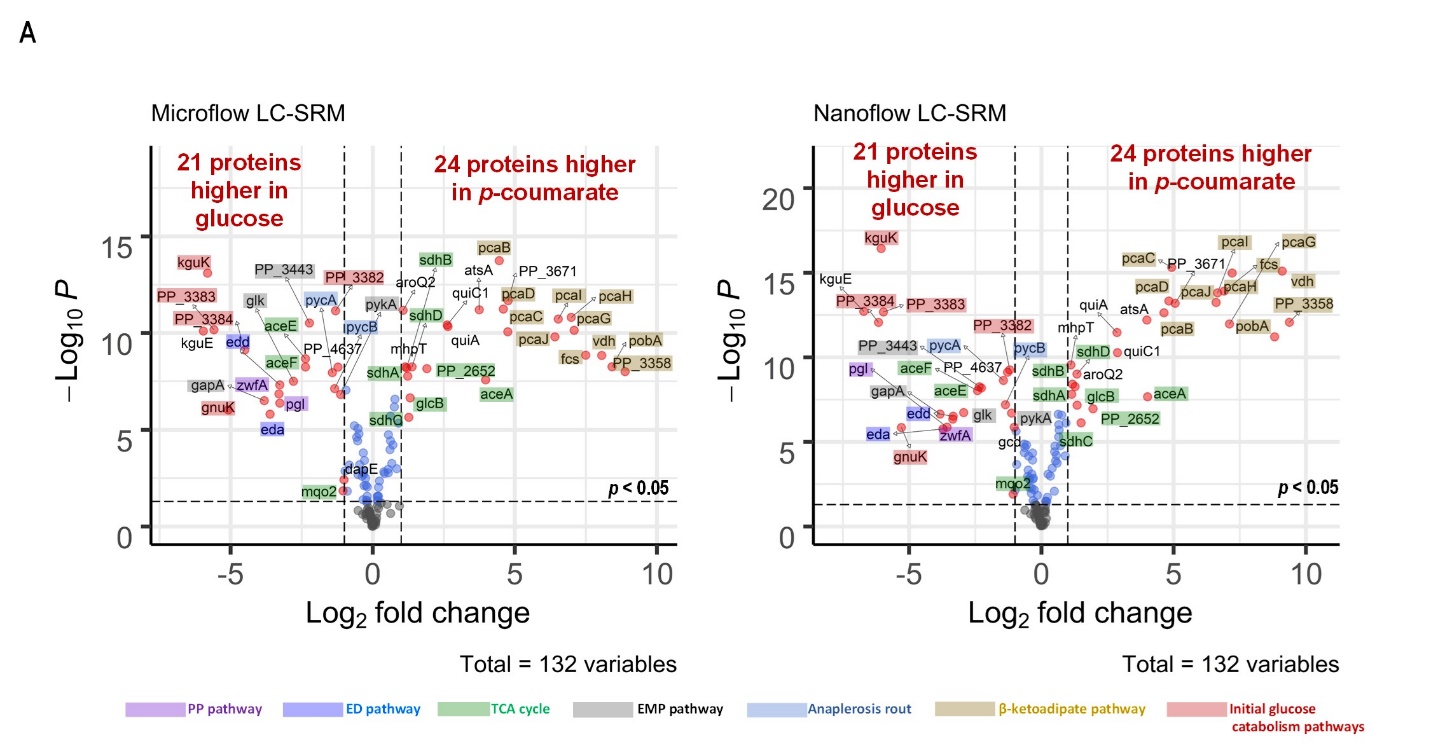


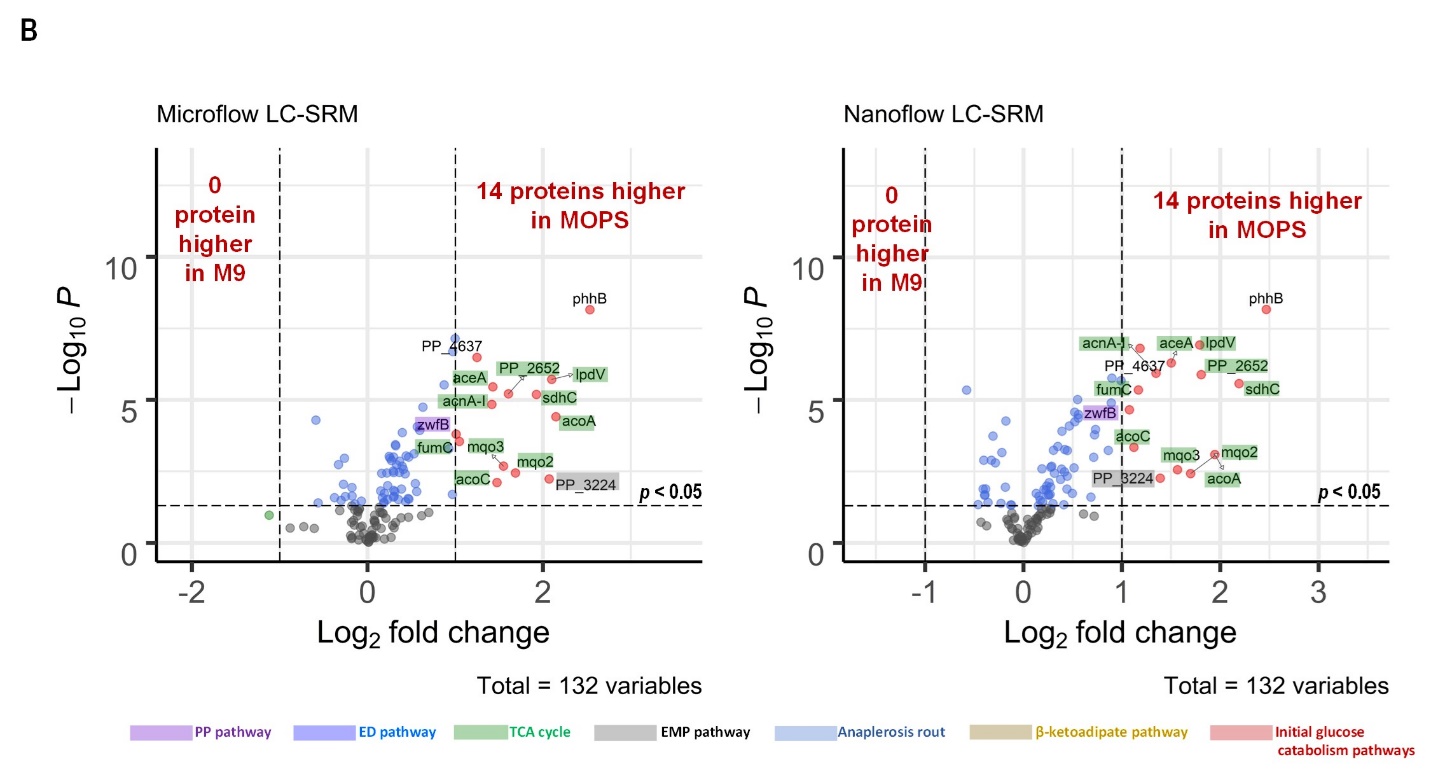


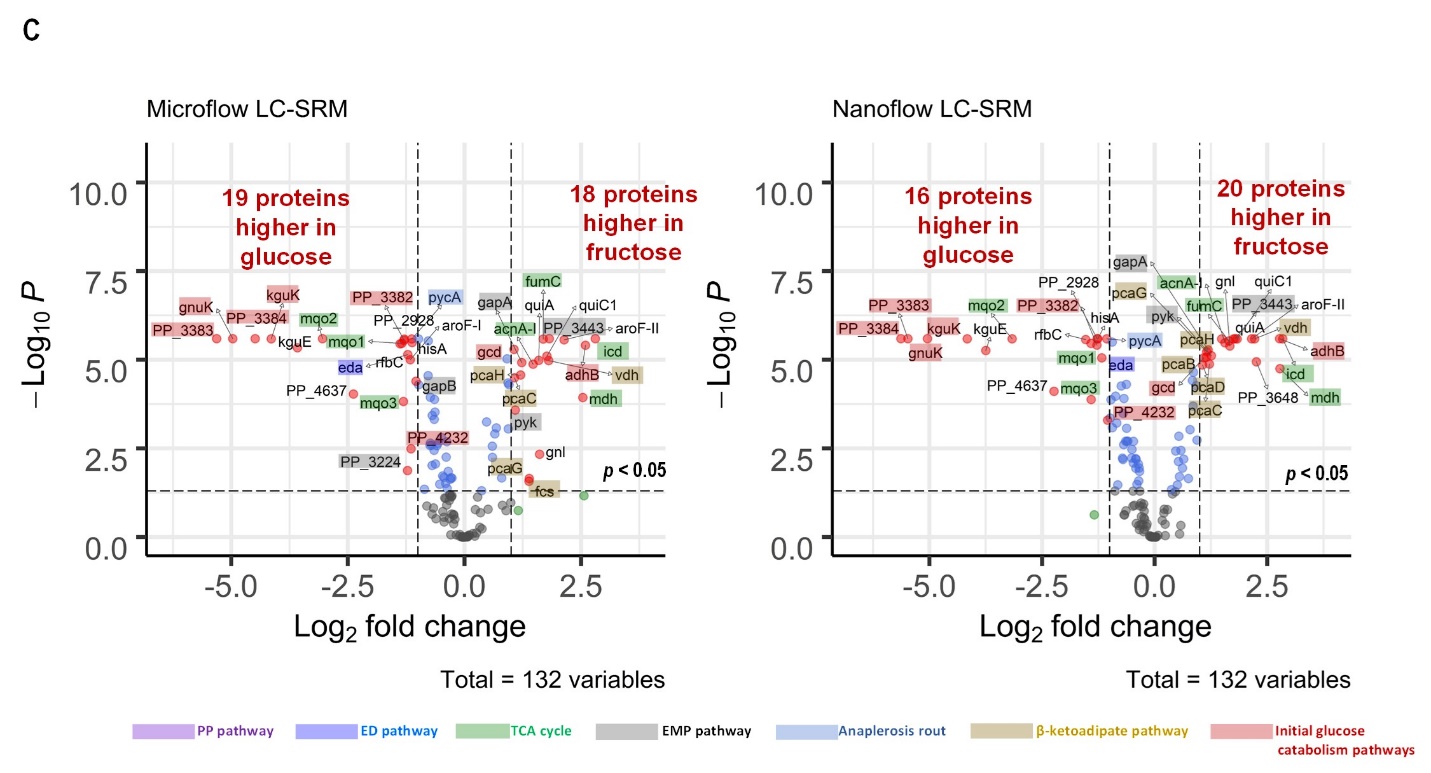


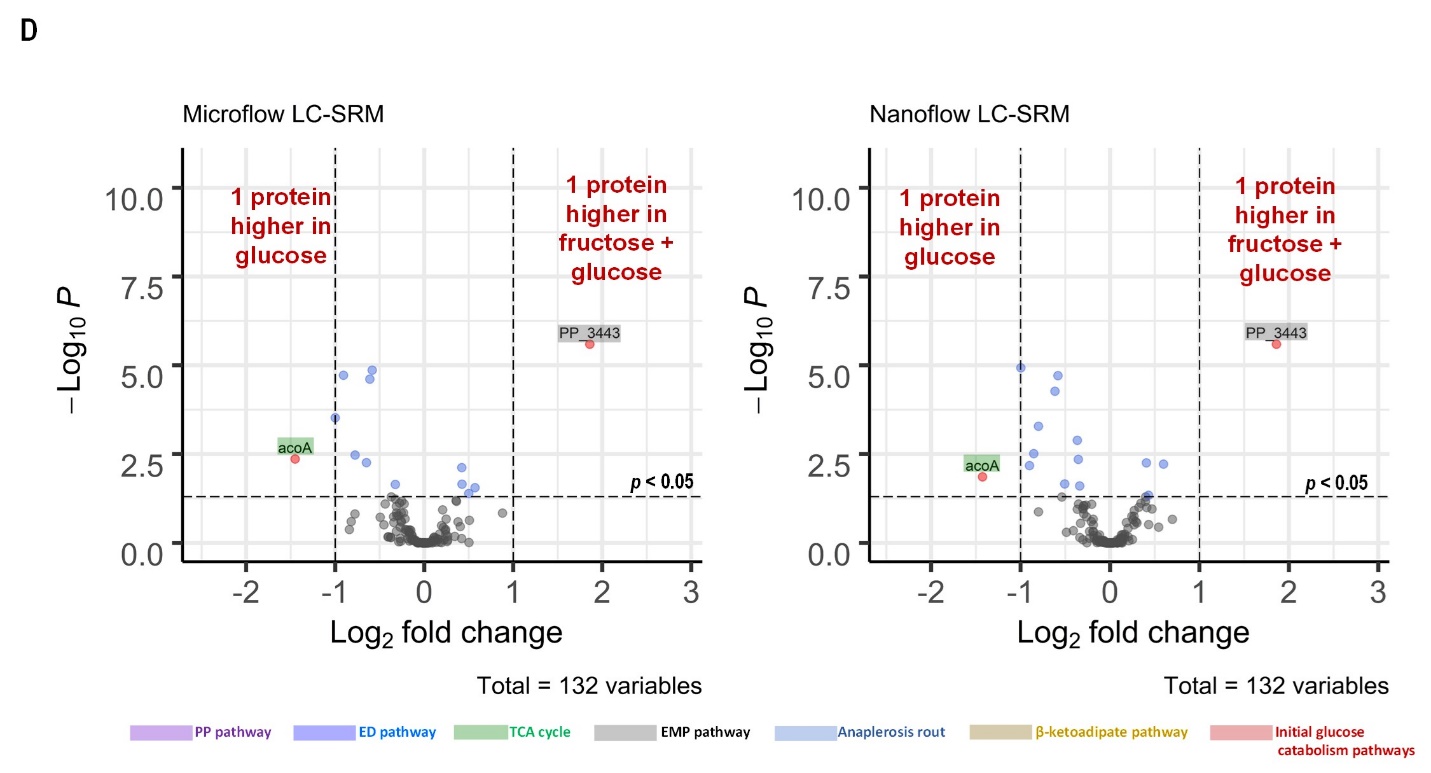

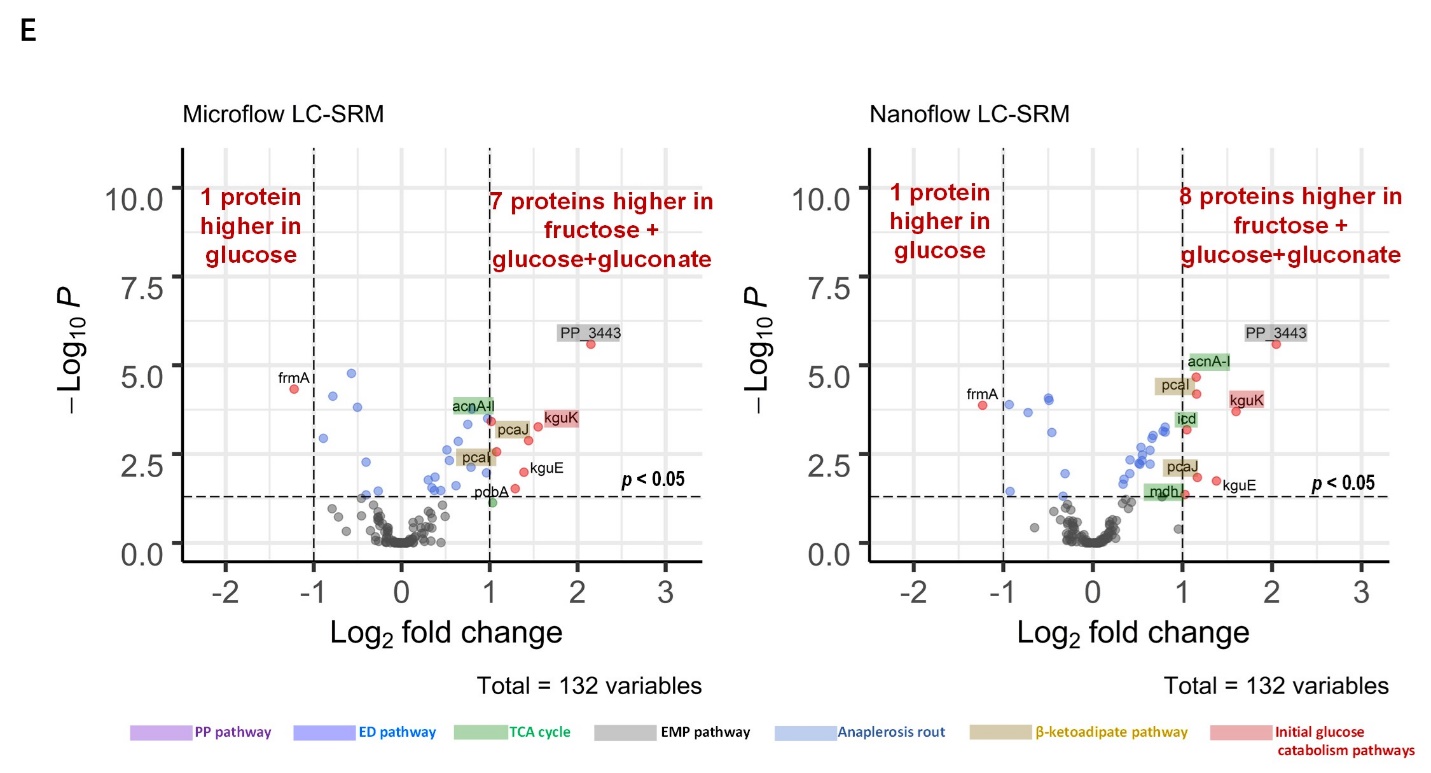


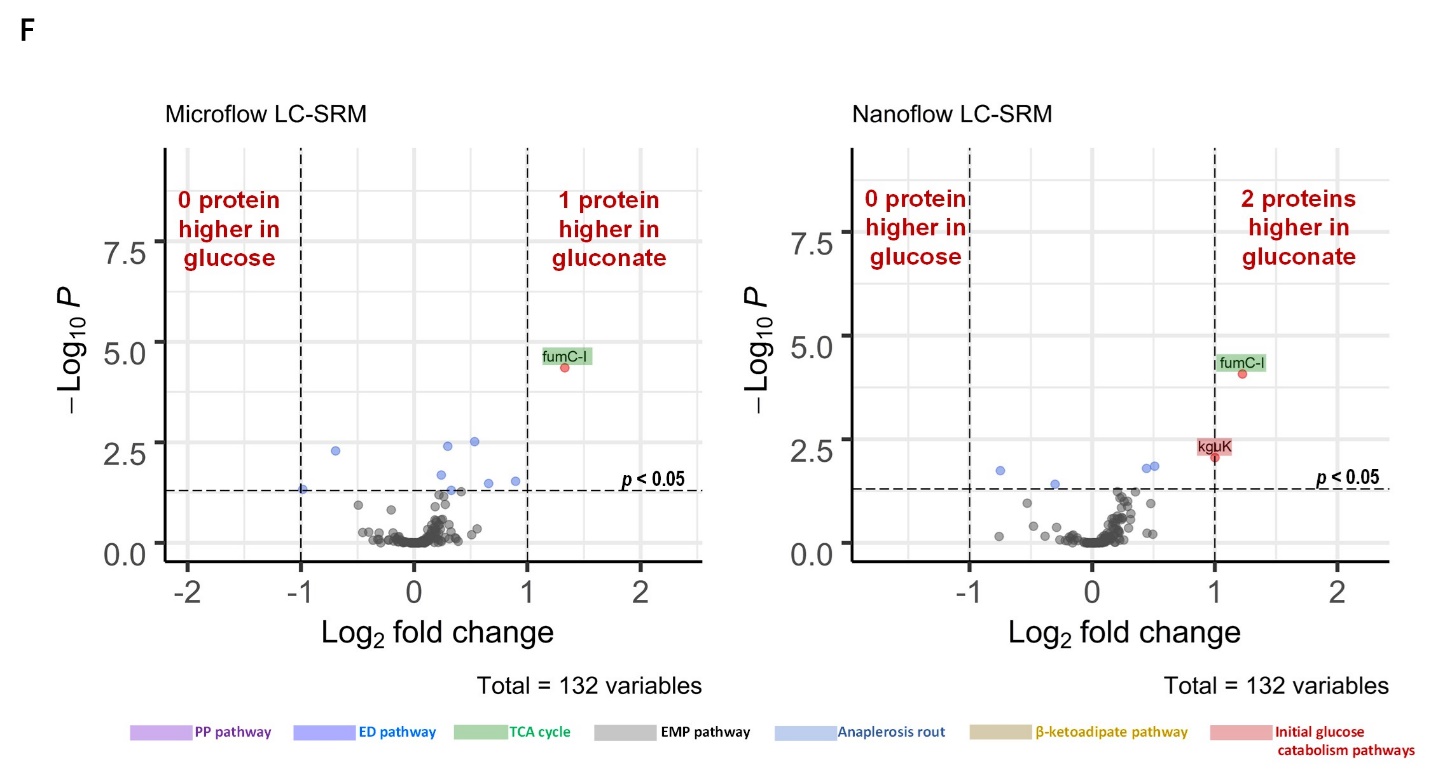


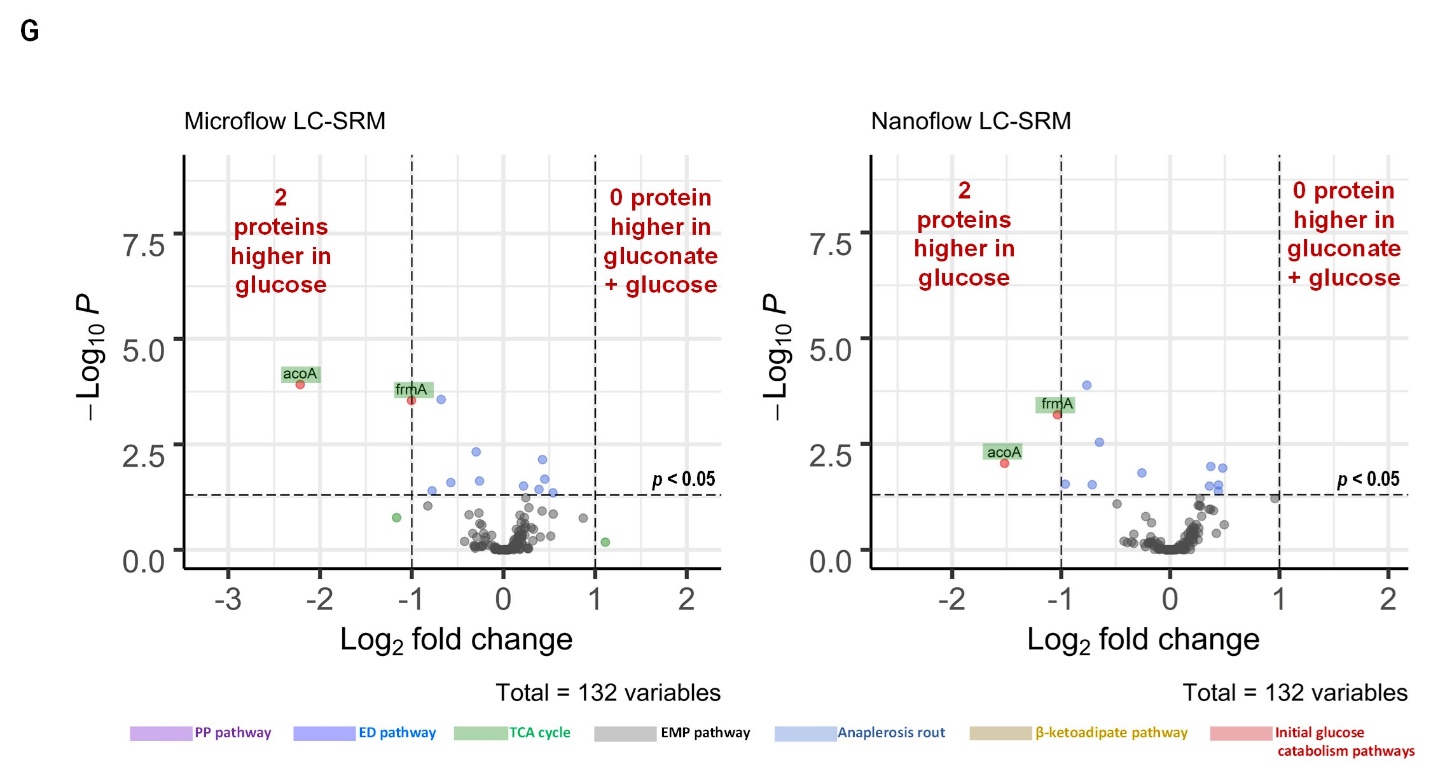


Supplementary Figure 4. Volcano plots displaying differential expressed genes in seven comparisons presented by both microflow LC-SRM and nanoflow LC-SRM. A) Compare strains grown in *p*-coumarate against those grown in glucose, both in MOPS medium. B) Compare strains grown in MOPS medium to those grown in M9 medium, both with glucose as carbon source. C) Compare strains grown in fructose against those grown in glucose, both in M9 medium. D) Compare strains grown in fructose+glucose against those grown in glucose, both in M9 medium. E) Compare strains grown in fructose+glucose+gluconate against those grown in glucose, both in M9 medium. F) Compare strains grown in gluconate against those grown in glucose, both in M9 medium. G) Compare strains grown in gluconate+gluocose against those grown in glucose, both in M9 medium. The vertical axis (y-axis) corresponds to the significance in terms of -log10(*p*-value), and the horizontal axis (x-axis) displays the log2 fold change value. The red dots represent significantly differentially expressed genes (*p*-value < 0.05, |fold change| > 2) that are either increased (right) or decreased (left); the blue dots represent the genes whose fold change is less than 2 folds in either direction but with enough significance (*p*-value < 0.05); the green dots represent genes whose fold change is more than 2 folds in either direction without enough significance (*p*-value > 0.05); the black dots represent genes whose fold change is less than 2 folds in either direction without enough significance (*p*-value > 0.05). All the significantly differentially expressed genes are label with their gene names. The total of variables plotted contain results of 132 genes. The shades on the gene labels indicate their pathway categories, including the Embden-Meyerhof-Parnas (EMP) pathway of glycolysis, pentose phosphate (PP) pathway, Entner-Doudoroff (ED) pathway, anaplerosis routes, tricarboxylic acid cycle (TCA), the initial glucose catabolism pathways, and β-ketoadipate pathway. Note: *fg* is short for fold change.


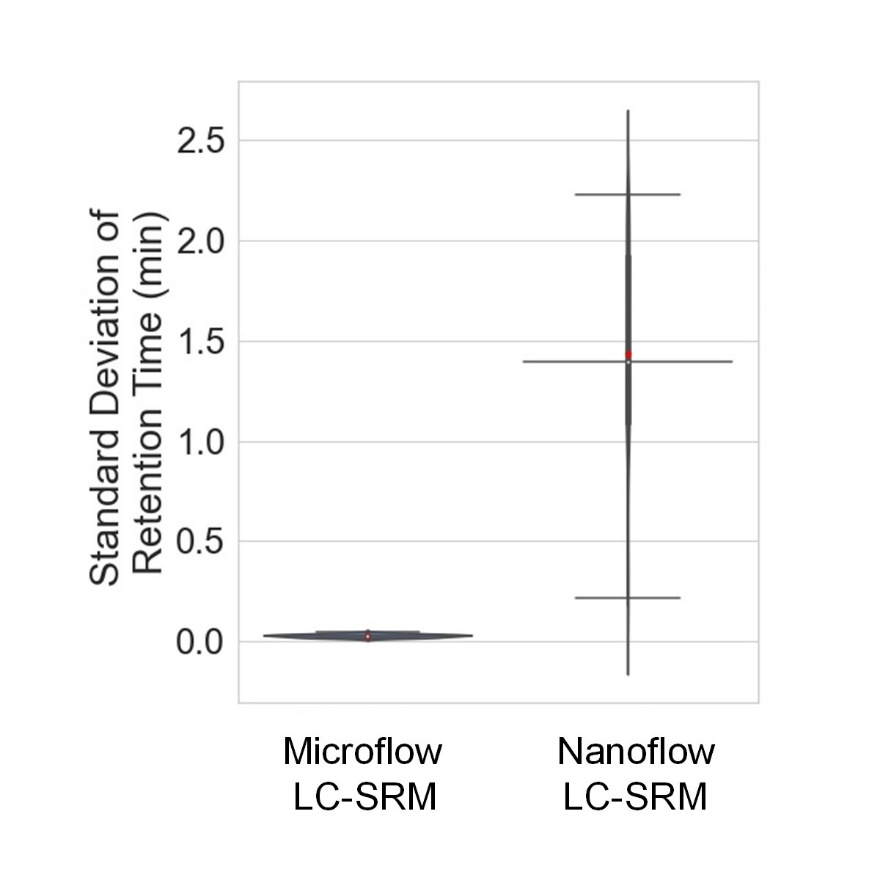


Supplementary Figure 5. Violin plot comparing the standard deviations of peptide retention time of 339 peptides monitored in 30 individual *P. putida* samples using either the microflow LC-SRM platform or nanoflow LC-SRM platform.


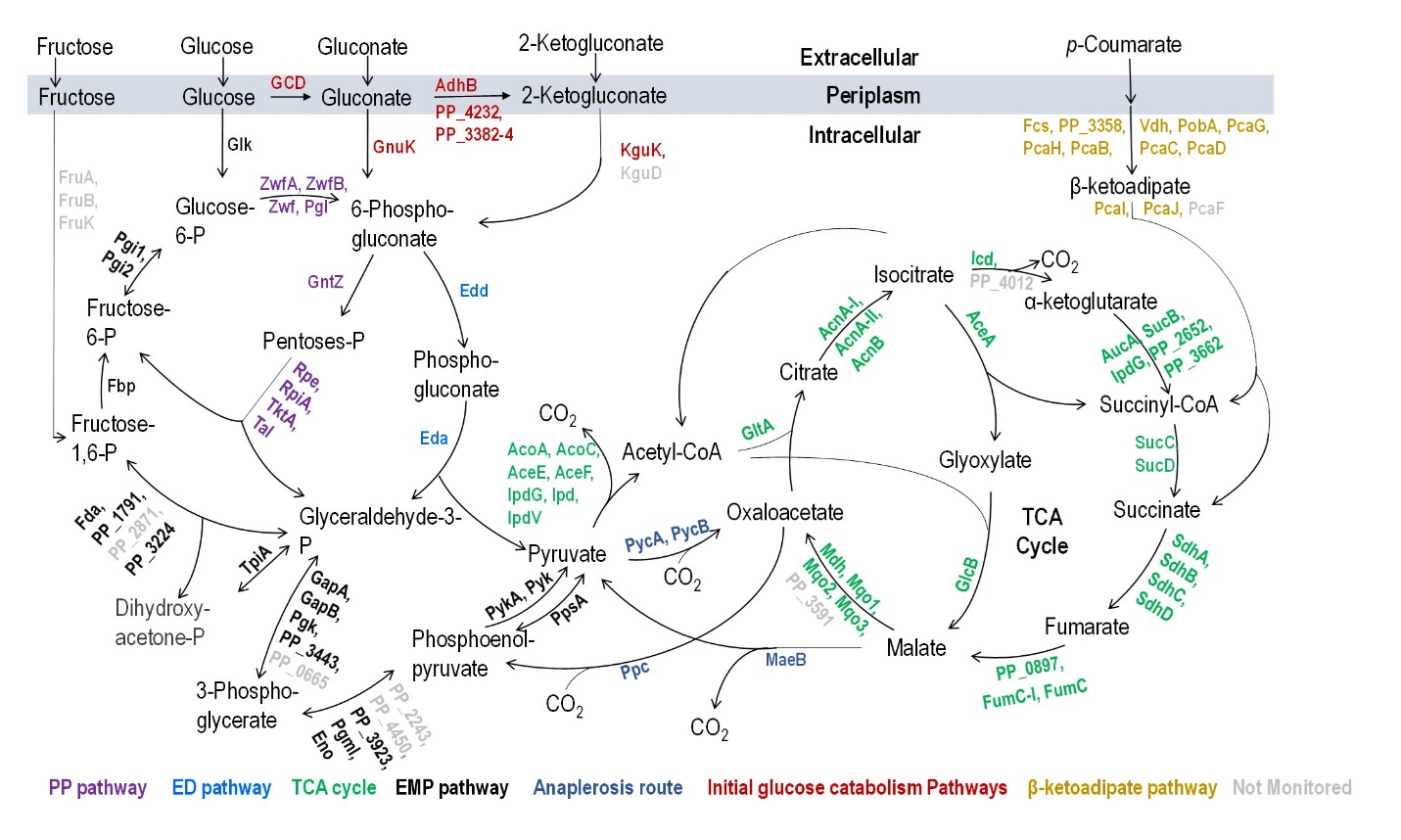


Supplementary Figure 6. Biochemical pathways involved in glucose, gluconate, fructose, p-coumarate catabolism in *P. putida* KT2440. Glucose and gluconate are first transferred into the periplasmic space and then into the cytoplasm. They are the Embden-Meyerhof-Parnas (EMP) pathway of glycolysis, pentose phosphate (PP) pathway, Entner-Doudoroff (ED) pathway, anaplerosis routes, tricarboxylic acid cycle (TCA), the initial glucose catabolism pathways, and β-ketoadipate pathway. The pathways are colour coded as shown in the legend. The genes in light grey color are pursued in this study.


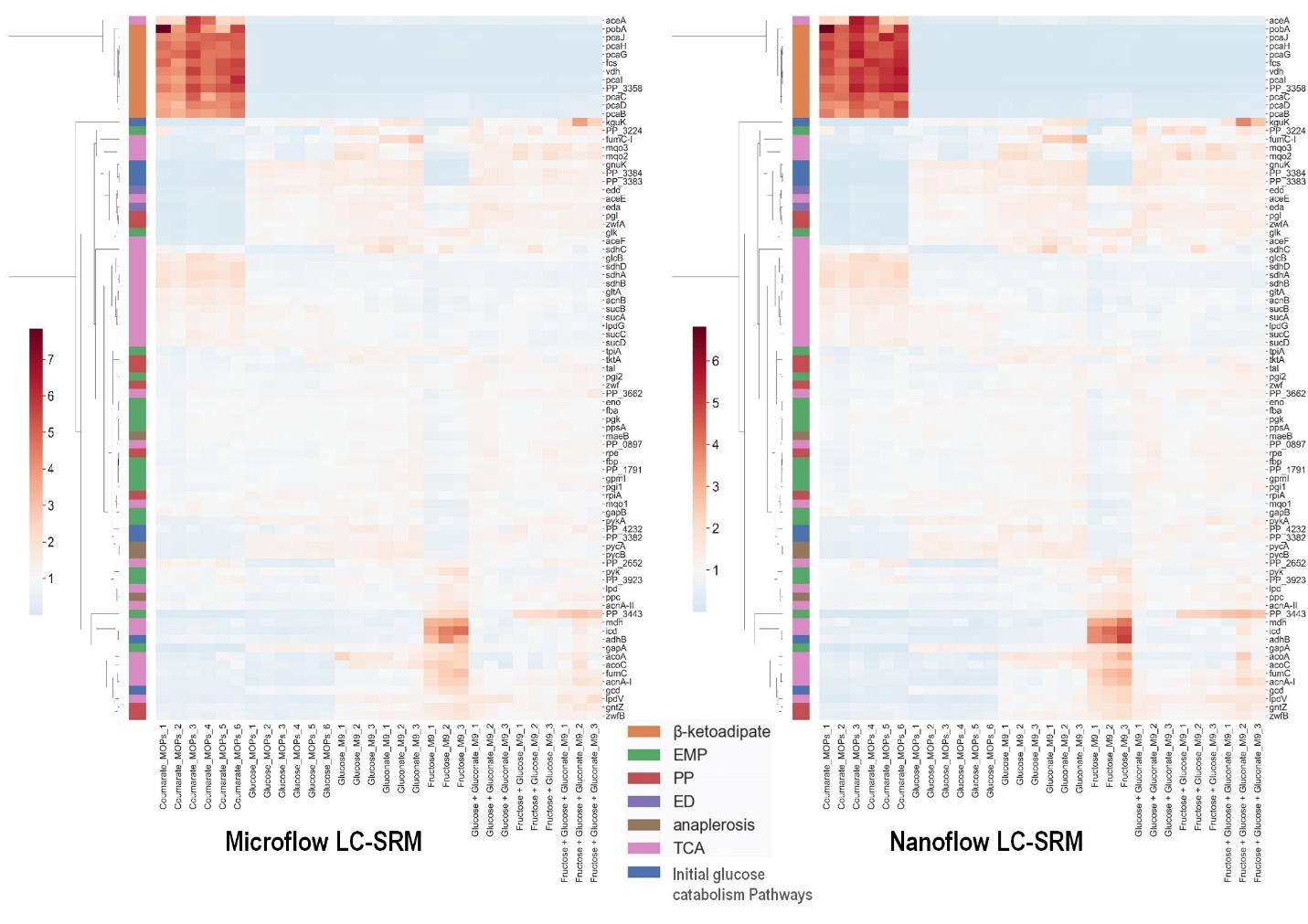


Supplementary Figure 7. Unsupervised hierarchical clustering of all the enzymes in the carbon catabolism in *P. putida* KT2440 based on the correlation coefficients between their protein expression level. There is clear separation of enzymes of β-ketoadipate pathway from the rest of the enzymes. The differential expression under various growth conditions show great similarity between microflow LC-SRM and nanoflow LC-SRM.
